# Deficiency of terminal complement pathway inhibitor promotes neuronal tau pathology and degeneration in mice

**DOI:** 10.1186/1742-2094-9-220

**Published:** 2012-09-18

**Authors:** Markus Britschgi, Yoshiko Takeda-Uchimura, Edward Rockenstein, Hudson Johns, Eliezer Masliah, Tony Wyss-Coray

**Affiliations:** 1 Department of Neurology and Neurological Sciences, Stanford University School of Medicine, 1201 Welch Road; MSLS Bldg, Rm P208, Stanford, CA 94305-5489, USA; 2 Departments of Neurosciences and Pathology, University of California San Diego, 9500 Gilman Drive # 9127, La Jolla, CA 92093-9127, USA; 3 Center for Tissue Regeneration, Repair and Restoration, Veterans Affairs Palo Alto Health Care System, 3801 Miranda Ave, Palo Alto, CA 94304, USA

**Keywords:** Age-related neurodegeneration, Alzheimer’s disease, Complement system, Frontotemporal lobar degeneration, Innate immune system, Mouse models of tau pathology, Tauopathy

## Abstract

**Background:**

The neuronal microtubule-associated protein tau becomes hyperphosphorylated and forms aggregates in tauopathies but the processes leading to this pathological hallmark are not understood. Because tauopathies are accompanied by neuroinflammation and the complement cascade forms a key innate immune pathway, we asked whether the complement system has a role in the development of tau pathology.

**Findings:**

We tested this hypothesis in two mouse models, which expressed either a central inhibitor of complement or lacked an inhibitor of the terminal complement pathway. Complement receptor-related gene/protein y is the natural inhibitor of the central complement component C3 in rodents. Expressing a soluble variant (sCrry) reduced the number of phospho-tau (AT8 epitope) positive neurons in the brain stem, cerebellum, cortex, and hippocampus of aged P301L mutant tau/sCrry double-transgenic mice compared with tau single-transgenic littermates (JNPL3 line). CD59a is the major inhibitor of formation of the membrane attack complex in mice. Intrahippocampal injection of adeno-associated virus encoding mutant human P301L tau into *Cd59a−/−* mice resulted in increased numbers of AT8-positive cells compared with wild-type controls. This was accompanied by neuronal and synaptic loss and reduced dendritic integrity.

**Conclusions:**

Our data in two independent mouse models with genetic changes in key regulators of the complement system support the hypothesis that the terminal pathway has an active role in the development of tau pathology. We propose that inhibition of the terminal pathway may be beneficial in tauopathies.

## Findings

Intraneuronal insoluble deposits of the microtubule-associated protein tau are found in neurodegenerative diseases commonly known as tauopathies [1]. One of the causes leading to these deposits in sporadic tauopathies may be aberrant phosphorylation of tau. A common feature in Alzheimer’s disease (AD), the most prevalent tauopathy, and other tauopathies is activation of immune pathways in the brain. The complement system is a key innate immune pathway, which is fully expressed in the brain, independent of peripheral contribution, and exerts critical homeostatic cerebral functions in development and aging (for extensive discussions and citations of relevant original articles about the role of complement system in the brain see [2,3]). Brains of patients with Pick’s disease (a pure tauopathy), AD, or individuals with Down’s syndrome with AD-pathology are found to have tangle-bearing neurons that are decorated with complement proteins, including the membrane attack complex (MAC). The presence of MAC in the brain even at early stages of AD or the deposition of products of complement activation in aged normal brains [4] suggests a lack of proper inhibitory control of the cascade with age and disease. Indeed, in affected brain regions of AD patients, levels of the main inhibitor of the MAC, CD59, are reduced [5,6]. These histological and biochemical findings in human beings open the question whether complement activation and formation of the MAC, in particular, are involved in the development of tau pathology.

**Figure 1:**
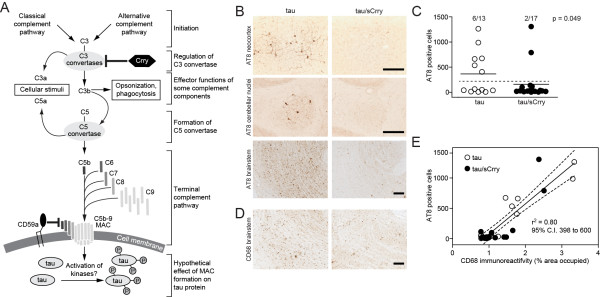
**Abnormal tau phosphorylation is reduced in genetically complement-inhibited P301L tau transgenic mice. A**, Simplified illustration of activation of the complement cascade in mice and its hypothetical effect on tau phosphorylation in the brain. Spontaneous (alternative pathway) or antibody-mediated (classical pathway) activation can initiate the complement cascade in the aging brain and if insufficiently controlled by inhibitory proteins (for example, Crry on C3 convertases or CD59a in the terminal pathway in mice) this can lead to the formation of a self-integrating membrane pore formed by C5b-9, also known as the membrane attack complex (MAC). The MAC disrupts cellular homeostasis by sublytic or lytic mechanisms potentially leading to activation of kinases, which could contribute to phosphorylation of tau protein. **B**, Examples of high and low tau pathology in various brain regions of 15 to 20 months aged P301L tau or P301L tau/sCrry transgenic mice, respectively, as detected by immunoreactivity of phospho-tau specific antibody AT8. **C**, Number of AT8-positive cells in the brainstem of 15 to 20 months aged tau (*n* = 13) and tau/sCrry (*n* = 17) transgenic mice. Each symbol indicates the mean from 5 to 6 sections per mouse (solid line is the group mean). The proportion of mice that developed tau pathology above the overall mean (dashed line) was significantly higher in tau mice than in the complement-inhibited tau/sCrry transgenic mice (2×2 contingency table, two-tailed Fisher’s exact test). **D**, Immunoreactivity for microglia marker CD68 in the brainstem of the two mice shown in B. **E**, Average number of AT8-positive cells in the brainstem plotted against % area of CD68 immunoreactivity in the same brain area. Linear regression with trend line (solid line) and 95% confidence intervals (C.I., dashed lines) are indicated; r^2^ as goodness-of-fit is significant (95% C.I. for the slope and *P* = 0.0001, two-tailed F test). Scale bar, 100 μm.

To test this hypothesis we crossed human mutant P301L tau transgenic mice (line JNLP3, herein called ‘tau transgenic’, a model for a genetic form of a pure tauopathy [7]; all described mouse experiments have been approved by the Palo Alto Veterans Hospital Institutional Review Board for Animal Experiments) with transgenic mice overexpressing a soluble form of the murine complement receptor 1-related gene/protein y (sCrry) [8] (Figure 1A). Tau transgenic (*n* = 13) and double-transgenic tau/sCrry mice (*n* = 17) were aged until the first mice started to present with the described lack of hind limb splay or weight loss in this line [7]. The symptoms developed, however, only in very few mice and only after 15 to 20 months, which was much slower and later in life than reported for the original line on a mixed genetic background. Mice were sacrificed and 30 μm floating cryotome brain sections were stained according to the mouse-on-mouse protocol (Vector Labs) with the phospho-tau specific antibody AT8 (0.5 μg/ml, mouse monoclonal antibody; Pierce Thermo Scientific). After developing with immunoperoxidase and diaminobenzidine, the number of AT8-positive cell bodies was counted blindly by two independent observers using light microscopy. AT8 immunoreactivity was observed in the neocortex, deep cerebellar nuclei, and, most prominently, brainstem but only in about one-third of all mice (Figure 1B). Despite this lack of consistent penetration of tau pathology, the proportion of mice that developed tau pathology in brainstem was significantly higher in tau transgenic mice (6/13) compared with the complement-inhibited tau/sCrry transgenic mice (2/17) (Figure 1C; two-tailed Fisher’s exact test *P* = 0.049). An increased inter-mouse variability of tau pathology has been described previously for the JNLP3 line after crossing it for several generations onto the C57BL/6 background [9]. Here, tau and tau/sCrry transgenic mice had a mixed background with at least 70% calculated contribution of the C57BL/6 J background. Neuroinflammation was quantified by relative staining intensity of microglial marker CD68 (diluted 1:50, FA-11; Serotec) in the same brain region. CD68 staining intensity correlated highly with the abundance of AT8-positive cells (Figure 1D,E). Together, this points to an involvement of innate immune pathways and in particular the complement system in tau pathology.

**Figure 2:**
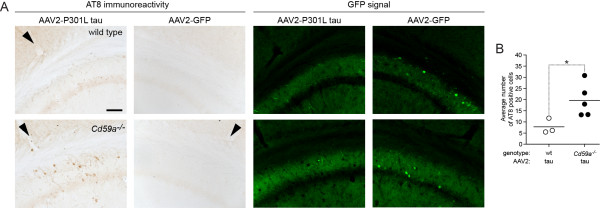
**Lack of CD59a promotes abnormal tau phosphorylation, which is accompanied by neuronal and synaptic degeneration. A**, Immunoreactivity of AT8 phosphorylated tau in the granular cell layer of the CA2/3 region of *Cd59a−/−* mice (*n* = 5) and wild-type (wt) littermate controls (*n* = 3) 5 months after intrahippocampal injection of adeno-associated virus (AAV2) encoding human P301L mutant tau (arrowheads indicates needle track). Contralateral hippocampus was injected with AAV2 encoding green fluorescent protein (AAV2-GFP), resulting in cytoplasmic and neuritic distribution of GFP. Occasionally, GFP-positive neuritis can also be observed in the AAV2-P301L tau-injected hemisphere. **B**, Number of AT8-positive cell bodies in the entire AAV2-P301L tau-injected hippocampus in *Cd59a−/−* mice and wild-type littermate controls. Each symbol indicates the mean number of 3 to 4 sections per mouse. Significance was calculated by Mann–Whitney U test; *P ≤ 0.05. Scale bar, 100 μm.

**Figure 3:**
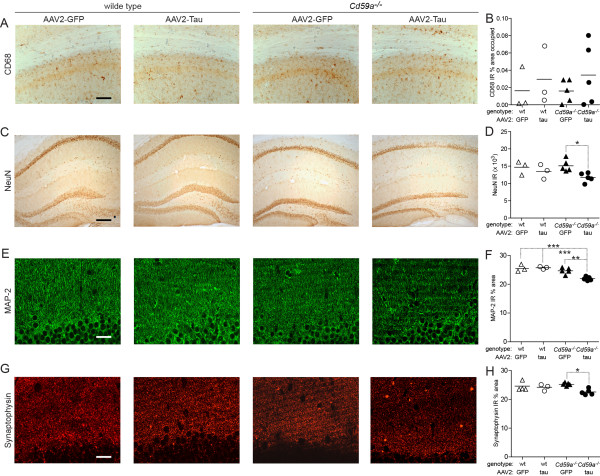
**Neuronal and synaptic degeneration in AAV2-P301L tau-injected hemisphere.** Microglial marker (**A,B**, CD68), neuronal (**C,D**, NeuN), dendritic (**E,F**, MAP-2), and synaptic (**G,H**, synaptophysin) integrity was quantified based on mean % area of immunoreactivity (IR, for example, neuropil occupied for the respective markers) (**B,F,H**) or IR alone (**D**). MAP-2 was quantified lateral to injection site to avoid overlap with the GFP signal. Each symbol indicates the mean number of 3 to 4 sections per mouse. Significance was calculated by ANOVA and post-hoc Tukey-Kramer test; *P ≤ 0.05, **P ≤ 0.01, ***P ≤ 0.001. Scale bar, 100 μm (**A**), 200 μm (**B**), and 50 μm (**D,E**).

Insertion of the MAC into the cell membrane in mice is tightly controlled by CD59a in mice and in human beings by CD59 [10] (Figure 1A). A lack thereof leaves cells more susceptible to spontaneous and induced attack by the MAC [10,11]. To test the effect of CD59a-deficiency on tau phosphorylation in the brain, *Cd59a−/−* mice (*n* = 5) [11] and wild-type littermates (*n* = 3) aged 4.5 months were injected stereotaxically with an adeno-associated virus (AAV2 serotype) encoding human P301L mutant tau in the right hippocampus and with an AAV2 encoding green fluorescent protein (GFP, internal control) in the left hippocampus. Two microliters of each AAV2 (tau or GFP; 8 × 10^12^ vg/ml [12]) were injected with a 10 μl Hamilton syringe for 2 min at a rate of 1 μl/min in a nanoinjector system. The needle was allowed to remain in the brain for an additional 2 min. The coordinates were anterior/posterior from bregma −2.0 mm, lateral +/− 1.5 mm, and dorsal/ventral 1.4 mm. After 5 months, mice were sacrificed and prepared for histological analysis as described previously. We observed a strong cytoplasmic and neuritic GFP signal in the granular layer of AAV2-GFP injected hippocampi of wild-type and *Cd59−/−* mice, consistent with robust expression of GFP and efficient transduction by AAV2. Occasionally, GFP-positive neurites were also present in the contralateral hemisphere (Figure 2A). The hippocampus of *Cd59a−/−* mice expressing human P301L mutant tau had significantly higher levels of AT8-positive cells than the respective hippocampus of their wild-type littermates (Figure 2A,B; *P* = 0.036, Mann–Whitney U test). AT8 immunoreactivity was detected in neuronal cell bodies, neurites, and axons in the CA2 and CA3 regions of the tau-injected side (Figure 2A). Relative staining intensity of microglial marker CD68 did not correlate with immunoreactivity for tau phosphorylation (Figure 3A,B). Tau phosphorylation was, however, accompanied by significant reduction of hippocampal immunoreactivity for markers of neuronal (NeuN and microtubule-associated protein 2, MAP-2; both antibodies at 1:500, Millipore) and synaptic integrity (synaptophysin, 1:500, Millipore) (Figure 3C-H; ANOVA and *post-hoc* Tukey-Kramer test). In conclusion, lack of CD59a promotes abnormal tau phosphorylation and loss of neuronal and synaptic integrity in mouse brains producing human P301L mutant tau pointing to an active role of the terminal complement pathway in tau pathology.

Earlier studies in mouse models of tauopathies reported a strong link between microglia activation and development of tau pathology [13-17] or vice versa [18]. Whereas some identified this link after administration of an exogenous trigger of inflammation [14-16], we observed a significant correlation between microglial activation and the number of AT8-positive cells in tau transgenic mice in the absence of exogenous stimuli (Figure 1D,E), which is consistent with findings in the brains of AD patients [19].

A growing number of studies show that the complement system probably has multiple functions in normal and injured brain and this may be relevant for AD [2,3]. For instance, overexpression of sCrry or ablating C3 in APP transgenic mouse models of AD accelerated formation of amyloid-β plaques and neurodegeneration [20,21]. This apparently protective effector function of the central component of the complement cascade may involve the opsonization of plaques followed by clearance of amyloid (Figure 1A). In contrast, full activation of the complement system and the terminal or lytic pathway can lead to formation of the MAC with possibly detrimental consequences. If not properly controlled by CD59, the MAC can generate differently sized (lytic and sublytic) pores in the cell membrane. Such pores then lead to increased Ca^2+^ influx, which may trigger depolarization of the membrane and activation of kinases in the cell [22]. Thus, full complement activation involving the terminal pathway and MAC formation may be upstream in the activation of kinases, such as MAPK, PKC, JNK, or PI3K/AKT, which have been implicated in the regulation of tau phosphorylation. Cellular and *in vivo* experiments also demonstrate that MAC formation in neurons can induce seizures and excitotoxicity, which promote neurodegeneration [23]. Excitotoxicity and seizures have also been proposed to contribute to cognitive decline in AD mouse models and patients. Interestingly, the absence of tau protects APP transgenic and wild-type mice from excitotoxic insults and prevents behavioral deficits [24]. Whether MAC formation and excitotoxicity are linked through tau would need to be studied in more detail.

Neuropathological analyses in human beings and our *in vivo* data do indeed point to an active role of the terminal complement pathway in the development of tau pathology, a neuropathological hallmark of AD and other tauopathies. Intriguingly, recent genome-wide association studies identified independently genetic variants of complement receptor 1 (CR1/CD35) and clusterin to be associated with AD ([25], replicated by others: http://www.alzgene.org). CR1/CD35 is one of the human functional analogs of the murine Crry and clusterin, which is also known as apolipoprotein J or complement lysis inhibitor, acts just one step upstream of CD59. It is interesting that, out of all immune-function-related proteins, two key regulators of the central component and the terminal cascade seem to be genetically linked with AD. It remains to be shown whether variants of genes coding for complement components or regulators are associated with other tauopathies as well.

In summary, complement proteins are key components of the innate immune system and have been implicated in homeostasis as well as degeneration of the human brain. Our data in two independent mouse models with genetic alterations in complement regulatory proteins support the hypothesis that the terminal pathway has an active role in the development of tau pathology. We propose that inhibition of the terminal pathway and, more specifically, inhibition of the MAC may be beneficial in tauopathies.

## Abbreviations

AAV2: Adeno-associated virus serotype 2; AD: Alzheimer’s disease; CR1: Complement receptor 1; GFP: Green fluorescent protein; IR: Immunoreactivity; MAC: Membrane attack complex; MAP-2: Microtubule-associated protein 2; sCrry: Soluble complement receptor 1-related gene/protein y; wt: Wild-type.

## Competing interests

MB is currently a full-time employee of Roche/F. Hoffmann-La Roche Ltd., Basel, Switzerland. The data related to this manuscript were generated when MB was still at Stanford University and the manuscript was written when MB was already at Roche. The authors declare that they have no competing interests.

## Authors’ contributions

MB and TW-C designed research and wrote the paper; MB, YT-U, HJ, ER, and EM performed research; MB, EM, and TW-C analyzed data. All authors read and approved the final manuscript.
